# 2-Hy­droxy-7-nitro­cyclo­hepta-2,4,6-trien-1-one

**DOI:** 10.1107/S1600536813006594

**Published:** 2013-03-16

**Authors:** Krzysztof Lyczko, Monika Lyczko

**Affiliations:** a Institute of Nuclear Chemistry and Technology, Dorodna 16, 03-195 Warsaw, Poland

## Abstract

The title compound, also known as 7-nitro­tropolone, C_7_H_5_NO_4_, exists in the crystalline state as the 2-hy­droxy-7-nitro­cyclo­hepta-2,4,6-trien-1-one tautomer and not as 2-hy­droxy-3-nitro­cyclo­hepta-2,4,6-trien-1-one. The dihedral angle between the ring and the nitro group is 70.3 (2)°. In the crystal, neighbouring mol­ecules are linked into dimers by a pair of O—H⋯O hydrogen bonds. In addition, the crystal is stabilized by O⋯π [3.4039 (14) Å] and O⋯O [3.073 (2) Å] inter­actions.

## Related literature

For the structure of tropolone and 5-nitro­tropolone, see: Shimanouchi & Sasada (1973 ▶); Kubo *et al.* (2001 ▶), respectively. Structural data on other mono-substituted tropolones were reported by Derry & Hamor (1972 ▶); Berg *et al.* (1976 ▶); Tsuji *et al.* (1991 ▶); Kubo *et al.* (2001 ▶, 2006*a* ▶,*b* ▶, 2007**a* ▶,*b* ▶,c* ▶,*d* ▶). For the synthesis of nitro­tropolone, see Cook *et al.* (1954 ▶).
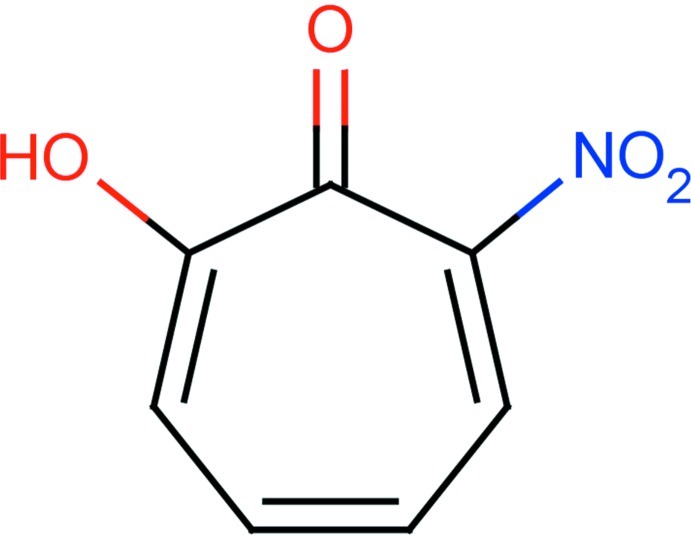


## Experimental

### 

#### Crystal data

C_7_H_5_NO_4_
*M* *_r_* = 167.12
Monoclinic, 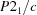

*a* = 9.6167 (2) Å
*b* = 6.4772 (1) Å
*c* = 11.7326 (4) Å
β = 96.162 (2)°
*V* = 726.59 (3) Å^3^
*Z* = 4
Cu *K*α radiation
μ = 1.11 mm^−1^
*T* = 295 K
0.45 × 0.35 × 0.15 mm

#### Data collection

Agilent SuperNova (Dual, Eos) diffractometer
Absorption correction: multi-scan (*CrysAlis PRO*; Agilent, 2010 ▶) *T*_min_ = 0.541, *T*_max_ = 1.000
8694 measured reflections
1364 independent reflections
1314 reflections with *I* > 2σ(*I*)
*R*_int_ = 0.022

#### Refinement

*R*[*F* ^2^ > 2σ(*F* ^2^)] = 0.038
*wR*(*F* ^2^) = 0.106
*S* = 1.07
1364 reflections
113 parameters
H atoms treated by a mixture of independent and constrained refinement
Δρ_max_ = 0.19 e Å^−3^
Δρ_min_ = −0.16 e Å^−3^

### 

Data collection: *CrysAlis PRO* (Agilent, 2010 ▶); cell refinement: *CrysAlis PRO*; data reduction: *CrysAlis PRO*; program(s) used to solve structure: *SHELXS97* (Sheldrick, 2008 ▶); program(s) used to refine structure: *SHELXL97* (Sheldrick, 2008 ▶); molecular graphics: *XP* in *SHELXTL* (Sheldrick, 2008 ▶) and *Mercury* (Macrae *et al.*, 2008 ▶); software used to prepare material for publication: *SHELXL97*.

## Supplementary Material

Crystal structure: contains datablock(s) global, I. DOI: 10.1107/S1600536813006594/kj2222sup1.cif

Structure factors: contains datablock(s) I. DOI: 10.1107/S1600536813006594/kj2222Isup2.hkl

Supplementary material file. DOI: 10.1107/S1600536813006594/kj2222Isup3.cml

Additional supplementary materials:  crystallographic information; 3D view; checkCIF report

## Figures and Tables

**Table 1:** Hydrogen-bond geometry (Å, °)

*D*—H⋯*A*	*D*—H	H⋯*A*	*D*⋯*A*	*D*—H⋯*A*
O2—H2⋯O1	0.84 (3)	2.05 (3)	2.5796 (14)	120 (2)
O2—H2⋯O1^i^	0.84 (3)	2.04 (3)	2.7349 (15)	139 (3)

Symmetry code: (i) 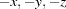.

**Fig. 1.**
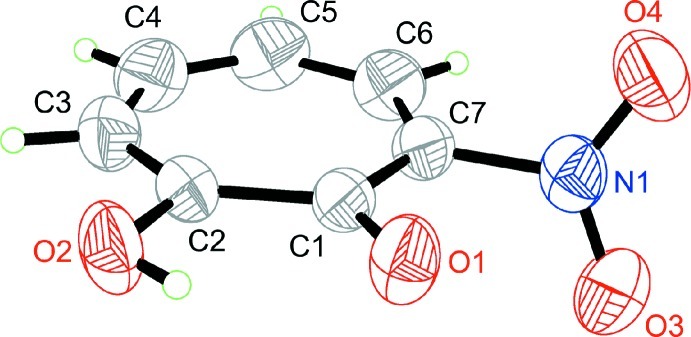
Molecular structure of the title compound. Displacement ellipsoids of the non-hydrogen atoms are drawn at the 50% probability level.

**Fig. 2.**
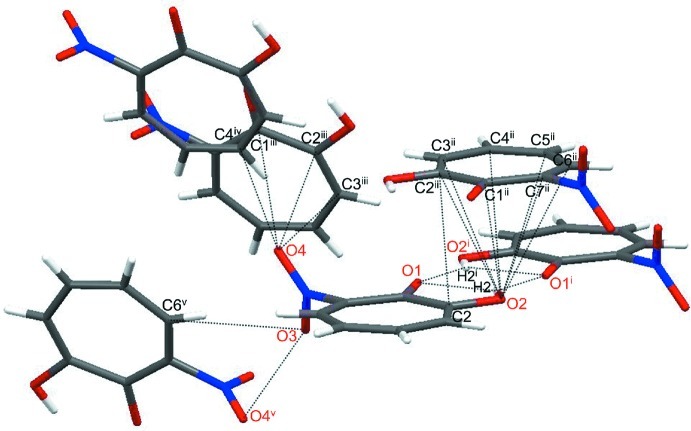
A fragment of the crystal structure showing the hydrogen bonds and intermolecular interactions in the 7-nitrotropolone. Dashed lines indicate hydrogen bonds and other intermolecular interactions.

## supplementary crystallographic information

### Comment

The aim of the present work was to determine the structure of the second isomer of nitrotropolone which is formed besides 5-nitrotropolone in the nitration reaction described by Cook *et al.* (1954). The second purpose was to find out which of the possible tautomers, 2-hydroxy-7-nitrocyclohepta- 2,4,6-trien-1-one or 2-hydroxy-3-nitrocyclohepta-2,4,6-trien-1-one is present in the solid state. It appeared that three different compounds were obtained during the nitration process with 7-nitrotropolone (Fig. 1) and 5-nitrotropolone as the main products and 7-hydroxytropolone as the side product.

In the title compound the C2—O2 bond (1.3324 (17) Å) is longer than the C1—O1 bond (1.2403 (16) Å). In combination with the features of the difference Fourier map this allows the unambiguous location of the hydroxyl H atom as bonded to the O2 atom. According to the rule which assigns position 1 in the tropolone ring to the carbon atom of the carbonyl group and position 2 to the carbon atom bonded to the hydroxyl group, the crystallized compound is 7-nitrotropolone and not 3-nitrotropolone.

The crystal structures of tropolone (Shimanouchi & Sasada, 1973) and other mono-substituted tropolones, such as 5-nitro-, 5-cyano- (Kubo *et al.*, 2001), 5-methyl- (Kubo *et al.*, 2007*b*), 5-methoxy- (Kubo *et al.*, 2006*b*), 5-acetoxy- (Kubo *et al.*, 2006*a*), 5-iodo- (Kubo *et al.*, 2007*d*), 7-iodo- (Kubo *et al.*, 2007*c*), 4-isopropyl- (Derry & Hamor, 1972), 5-isopropyl- (Berg *et al.*, 1976), 7-hydroxy- (Kubo *et al.*, 2007*a*), 7-chlorotropolone (Tsuji *et al.*, 1991), have been reported previously.

The studied compound forms centrosymmetric O—H···O hydrogen-bonded dimers, similar to those found for 5-nitrotropolone (Kubo *et al.*, 2001), tropolone (Shimanouchi & Sasada, 1973) and the most of its mono-substituted derivatives. Some of the other derivatives of tropolone, e.g. 4-isopropyltropolone (Derry & Hamor, 1972), 5-iodotropolone (Kubo *et al.*, 2007*d*) and 7-iodotropolone (Kubo *et al.*, 2007*c*), form hydrogen-bonded zigzag chains. The crystal structure of the title compound contains molecules linked into dimers through intermolecular O—H···O hydrogen-bonds involving the OH groups and the carbonyl O atoms (Table 1, Fig. 2). The hydroxyl group is in fact involved in a bifurcated hydrogen bond that also forms an intramolecular link to the carbonyl acceptor in the same molecule. The intermolecular distance O1···O2 of 2.7349 (15) Å is similar to that found for 5-nitrotropolone (2.743 Å, Kubo *et al.*, 2001) and tropolone (2.746 Å, Shimanouchi & Sasada, 1973). Intermolecular interactions between the hydroxyl group and a seven-membered ring (O—H···π interactions) are also observed; the O2···C(1–7)^ii^ [symmetry code: (ii) -*x*, 1 - *y*, -*z*] distances are in the range 3.585–3.637 Å. Furthermore, interactions between the nitro group and neighbouring rings of tropolone molecules (O···π interactions) are observed in the crystal structure: 3.149 Å for O4···C2^iii^, 3.321 Å for O4···C3^iii^, 3.472 Å for O4···C1^iii^ [symmetry code: (iii) *x*, 1/2 - *y*, 1/2 + *z*], 3.430 Å for O4···C4^iv^ [symmetry code: (iv) *x*, 3/2 - *y*, 3/2 + *z*] and 3.499 Å for O3···C6^v^ [symmetry code: (v) 1 - *x*, -1/2 + *y*, 1/2 - *z*]. The closest distance between symmetry related tropolone planes is 3.272 Å for C2···C2^ii^. However, the intermolecular π···π interactions between neighbouring rings are much less distinct in the crystal structure of 7-nitrotropolone than for 5-nitrotropolone (Kubo *et al.*, 2001). The shortest contact between oxygen atoms from neighbouring NO_2_ groups is 3.073 Å for O3···O4^v^. All of the interactions mentioned above have an influence on the formation of crystal structure network (Fig. 2). In 5-nitrotropolone (Kubo *et al.*, 2001) all atoms of the NO_2_ group lie exactly in (torsion angle C6—C5—N1—O4 0.2 (2)°) or very closely to (torsion angle C13—C12—N1—O7 13.7 (2)°) the plane of the tropolone ring while for the 7-nitrotropolone this group is rotated out of the tropolone plane (torsion angle C1—C7—N1—O3 71.5 (2)°).

### Experimental

The title compound and 5-nitrotropolone were synthesized together as the main products in the nitration reaction of tropolone described by Cook *et al.* (1954). 7-hydroxytropolone was also obtained in this reaction but in a much smaller amount. Single crystals of these compounds were found after recrystallization from benzene. The formation of these products were confirmed by single-crystal X-ray diffraction measurements.

### Refinement

The H atom of the OH group was located in a difference map and its position and *U*_iso_ value were freely refined. All H atoms bonded to C atoms were placed in calculated positions with C—H = 0.93 Å and refined isotropically using riding model with *U*_iso_(H) = 1.2 *U*_eq_(C).

### Crystal data

**Table:**

C_7_H_5_NO_4_	*F*(000) = 344
*M**_r_* = 167.12	*D*_x_ = 1.528 Mg m^−^^3^
Monoclinic, *P*2_1_/*c*	Cu *K*α radiation, λ = 1.54178 Å
Hall symbol: -P 2ybc	Cell parameters from 6294 reflections
*a* = 9.6167 (2) Å	θ = 3.8–72.1°
*b* = 6.4772 (1) Å	µ = 1.11 mm^−^^1^
*c* = 11.7326 (4) Å	*T* = 295 K
β = 96.162 (2)°	Plate, yellow
*V* = 726.59 (3) Å^3^	0.45 × 0.35 × 0.15 mm
*Z* = 4	

### Data collection

**Table:**

Agilent SuperNova (Dual, Eos) diffractometer	1364 independent reflections
Radiation source: SuperNova (Cu) X-ray Source	1314 reflections with *I* > 2σ(*I*)
Mirror monochromator	*R*_int_ = 0.022
Detector resolution: 16.0131 pixels mm^-1^	θ_max_ = 70.0°, θ_min_ = 7.6°
ω scans	*h* = −11→11
Absorption correction: multi-scan (*CrysAlis PRO*; Agilent, 2010)	*k* = −7→7
*T*_min_ = 0.541, *T*_max_ = 1.000	*l* = −14→13
8694 measured reflections	

### Refinement

**Table:**

Refinement on *F*^2^	Primary atom site location: structure-invariant direct methods
Least-squares matrix: full	Secondary atom site location: difference Fourier map
*R*[*F*^2^ > 2σ(*F*^2^)] = 0.038	Hydrogen site location: inferred from neighbouring sites
*wR*(*F*^2^) = 0.106	H atoms treated by a mixture of independent and constrained refinement
*S* = 1.07	*w* = 1/[σ^2^(*F*_o_^2^) + (0.0534*P*)^2^ + 0.1985*P*] where *P* = (*F*_o_^2^ + 2*F*_c_^2^)/3
1364 reflections	(Δ/σ)_max_ < 0.001
113 parameters	Δρ_max_ = 0.19 e Å^−^^3^
0 restraints	Δρ_min_ = −0.16 e Å^−^^3^

### Special details

**Table:**

Geometry. All e.s.d.'s (except the e.s.d. in the dihedral angle between two l.s. planes) are estimated using the full covariance matrix. The cell e.s.d.'s are taken into account individually in the estimation of e.s.d.'s in distances, angles and torsion angles; correlations between e.s.d.'s in cell parameters are only used when they are defined by crystal symmetry. An approximate (isotropic) treatment of cell e.s.d.'s is used for estimating e.s.d.'s involving l.s. planes.
Refinement. Refinement of *F*^2^ against ALL reflections. The weighted *R*-factor *wR* and goodness of fit *S* are based on *F*^2^, conventional *R*-factors *R* are based on *F*, with *F* set to zero for negative *F*^2^. The threshold expression of *F*^2^ > σ(*F*^2^) is used only for calculating *R*-factors(gt) *etc*. and is not relevant to the choice of reflections for refinement. *R*-factors based on *F*^2^ are statistically about twice as large as those based on *F*, and *R*- factors based on ALL data will be even larger.

### Fractional atomic coordinates and isotropic or equivalent isotropic displacement parameters (Å^2^)

**Table:**

	*x*	*y*	*z*	*U*_iso_*/*U*_eq_	
C1	0.16992 (13)	0.2812 (2)	0.03140 (11)	0.0391 (3)	
C2	0.10047 (15)	0.3639 (2)	−0.07541 (11)	0.0427 (3)	
C3	0.12800 (17)	0.5388 (2)	−0.13382 (13)	0.0530 (4)	
C4	0.22824 (19)	0.6924 (3)	−0.10795 (15)	0.0600 (4)	
C5	0.32680 (19)	0.7082 (3)	−0.01626 (16)	0.0613 (5)	
C6	0.35254 (16)	0.5684 (2)	0.07413 (14)	0.0535 (4)	
C7	0.28716 (13)	0.3865 (2)	0.09138 (11)	0.0416 (3)	
H2	−0.011 (3)	0.146 (4)	−0.077 (2)	0.101 (9)*	
H3	0.0707	0.5589	−0.2019	0.064*	
H4	0.2275	0.7992	−0.1610	0.072*	
H5	0.3833	0.8250	−0.0134	0.074*	
H6	0.4241	0.6046	0.1302	0.064*	
N1	0.34481 (12)	0.2721 (2)	0.19480 (11)	0.0500 (3)	
O1	0.12366 (11)	0.11924 (15)	0.06932 (9)	0.0534 (3)	
O2	−0.00635 (12)	0.24914 (19)	−0.12093 (9)	0.0568 (3)	
O3	0.40798 (16)	0.1136 (2)	0.18063 (12)	0.0853 (5)	
O4	0.32868 (16)	0.3421 (2)	0.28807 (10)	0.0751 (4)	

### Atomic displacement parameters (Å^2^)

**Table:**

	*U*^11^	*U*^22^	*U*^33^	*U*^12^	*U*^13^	*U*^23^
C1	0.0399 (7)	0.0356 (7)	0.0401 (7)	−0.0002 (5)	−0.0038 (5)	−0.0029 (5)
C2	0.0472 (7)	0.0425 (7)	0.0367 (7)	0.0006 (6)	−0.0031 (5)	−0.0048 (5)
C3	0.0655 (9)	0.0517 (9)	0.0404 (7)	0.0028 (7)	−0.0011 (6)	0.0057 (6)
C4	0.0757 (11)	0.0452 (9)	0.0603 (10)	−0.0017 (8)	0.0125 (8)	0.0114 (7)
C5	0.0628 (10)	0.0438 (8)	0.0777 (12)	−0.0129 (7)	0.0091 (8)	0.0015 (8)
C6	0.0463 (8)	0.0496 (8)	0.0626 (9)	−0.0102 (6)	−0.0036 (6)	−0.0074 (7)
C7	0.0379 (7)	0.0418 (7)	0.0433 (7)	0.0003 (5)	−0.0041 (5)	−0.0036 (5)
N1	0.0433 (7)	0.0532 (7)	0.0500 (7)	−0.0057 (5)	−0.0109 (5)	−0.0016 (5)
O1	0.0569 (6)	0.0433 (6)	0.0551 (6)	−0.0128 (5)	−0.0164 (5)	0.0074 (4)
O2	0.0653 (7)	0.0564 (7)	0.0433 (6)	−0.0133 (5)	−0.0188 (5)	0.0017 (5)
O3	0.0887 (10)	0.0850 (10)	0.0759 (9)	0.0384 (8)	−0.0202 (7)	0.0027 (7)
O4	0.1024 (10)	0.0742 (9)	0.0475 (7)	−0.0110 (7)	0.0025 (6)	−0.0021 (6)

### Geometric parameters (Å, º)

**Table:**

C1—C7	1.4358 (18)	C6—H6	0.9300
C2—C1	1.4575 (18)	C7—C6	1.361 (2)
C2—C3	1.364 (2)	N1—O3	1.2135 (19)
C3—H3	0.9300	N1—O4	1.2098 (17)
C4—C3	1.396 (2)	N1—C7	1.4779 (18)
C4—C5	1.359 (3)	O1—C1	1.2403 (16)
C4—H4	0.9300	O2—C2	1.3324 (17)
C5—H5	0.9300	O2—H2	0.84 (3)
C6—C5	1.396 (2)		
			
C1—C7—N1	111.71 (12)	C6—C7—C1	133.59 (14)
C2—O2—H2	106.8 (19)	C6—C7—N1	114.68 (12)
C2—C3—C4	130.48 (14)	C7—C1—C2	120.66 (12)
C2—C3—H3	114.8	C7—C6—C5	128.85 (15)
C3—C2—C1	129.91 (13)	C7—C6—H6	115.6
C3—C4—H4	115.4	O1—C1—C2	118.07 (12)
C4—C3—H3	114.8	O1—C1—C7	121.26 (12)
C4—C5—C6	127.17 (15)	O2—C2—C1	113.69 (12)
C4—C5—H5	116.4	O2—C2—C3	116.41 (13)
C5—C4—C3	129.22 (15)	O3—N1—C7	117.45 (13)
C5—C4—H4	115.4	O4—N1—C7	118.79 (13)
C5—C6—H6	115.6	O4—N1—O3	123.76 (15)
C6—C5—H5	116.4		
			
C1—C2—C3—C4	−1.6 (3)	O1—C1—C7—C6	−175.17 (15)
C1—C7—C6—C5	−4.0 (3)	O1—C1—C7—N1	3.12 (19)
C2—C1—C7—C6	3.9 (2)	O2—C2—C1—C7	179.96 (12)
C2—C1—C7—N1	−177.80 (11)	O2—C2—C1—O1	−0.93 (19)
C3—C2—C1—C7	−0.5 (2)	O2—C2—C3—C4	178.01 (16)
C3—C2—C1—O1	178.65 (15)	O3—N1—C7—C1	71.52 (17)
C3—C4—C5—C6	1.4 (3)	O3—N1—C7—C6	−109.84 (17)
C5—C4—C3—C2	0.2 (3)	O4—N1—C7—C1	−109.60 (15)
C7—C6—C5—C4	0.4 (3)	O4—N1—C7—C6	69.04 (18)
N1—C7—C6—C5	177.76 (16)		

### Hydrogen-bond geometry (Å, º)

**Table:**

*D*—H···*A*	*D*—H	H···*A*	*D*···*A*	*D*—H···*A*
O2—H2···O1	0.84 (3)	2.05 (3)	2.5796 (14)	120 (2)
O2—H2···O1^i^	0.84 (3)	2.04 (3)	2.7349 (15)	139 (3)

Symmetry code: (i) −*x*, −*y*, −*z*.
